# Selective targeting of bioengineered platelets to prostate cancer vasculature: new paradigm for therapeutic modalities

**DOI:** 10.1111/jcmm.12515

**Published:** 2015-03-04

**Authors:** Viviana P Montecinos, Claudio H Morales, Thomas H Fischer, Sarah Burns, Ignacio F San Francisco, Alejandro S Godoy, Gary J Smith

**Affiliations:** a Department of Hematology-Oncology, Pontificia Universidad Católica de ChileSantiago, Chile; b Department of Urology, Roswell Park Cancer InstituteBuffalo, NY, USA; c Hospital Clínico Dr. Félix Bulnes Cerda, Servicio de Salud Metropolitano OccidenteSantiago, Chile; d Department of Pathology and Laboratory Medicine, University of North Carolina at Chapel HillChapel Hill, NC, USA; e Department of Medicine, Roswell Park Cancer InstituteBuffalo, NY, USA; f Department of Urology, Pontificia Universidad Católica de ChileSantiago, Chile; g Department of Physiology, Pontificia Universidad Católica de ChileSantiago, Chile

**Keywords:** platelets, androgen deprivation, human prostate xenografts, SPIO nanoparticles

## Abstract

Androgen deprivation therapy (ADT) provides palliation for most patients with advanced prostate cancer (CaP); however, greater than 80% subsequently fail ADT. ADT has been indicated to induce an acute but transient destabilization of the prostate vasculature in animal models and humans. Human re-hydrated lyophilized platelets (hRL-P) were investigated as a prototype for therapeutic agents designed to target selectively the tumour-associated vasculature in CaP. The ability of hRL-P to bind the perturbed endothelial cells was tested using thrombin- and ADP-activated human umbilical vein endothelial cells (HUVEC), as well as primary xenografts of human prostate tissue undergoing acute vascular involution in response to ADT. hRL-P adhered to activated HUVEC in a dose-responsive manner. Systemically administered hRL-P, and hRL-P loaded with super-paramagnetic iron oxide (SPIO) nanoparticles, selectively targeted the ADT-damaged human microvasculature in primary xenografts of human prostate tissue. This study demonstrated that hRL-P pre-loaded with chemo-therapeutics or nanoparticles could provide a new paradigm for therapeutic modalities to prevent the rebound/increase in prostate vasculature after ADT, inhibiting the transition to castration-recurrent growth.

## Introduction

The ability of a cancer to increase vascular density within the tumour mass is requisite for growth beyond a small nodule 1. During this angiogenic switch, endothelial cells respond to hypoxia and changes in the local environment, and migrate towards the tumour forming new blood vessels that facilitate tumour growth and metastasis. However, four decades after Dr. Judah Folkman introduced the concept of anti-angiogenic therapy as an exciting anti-cancer treatment modality, and after hundreds of clinical trials, anti-angiogenic treatment regimes have produced only modest objective responses with limited duration in cancer patients 2,3. Therefore, new strategies are needed to selectively destabilize tumour vasculature as a mechanism to affect cancer cell viability.

In prostate adenocarcinoma (CaP), increased microvessel density (MVD) in tumour tissue has been correlated with increased tumour stage, tumour grade, metastasis and decreased cancer-specific survival 4–6. Androgen deprivation therapy (ADT), the standard-of-treatment for advanced or metastatic CaP for over 70 years, provides transient palliation for most men, however, as a result of this therapy, CaP progresses from an androgen-sensitive phenotype to the more aggressive, and eventually lethal, castration-recurrent phenotype. Even though the transition to the castration-recurrent phenotype is associated with an increase in MVD within the human prostate cancer tissue 4,5, paradoxically, ADT induces initially an acute reduction in prostate MVD as measured by quantitation of endothelial cells 7,8. Using a unique pre-clinical model of primary xenografts of intact human prostate tissue transplanted to immunocompromised mice supplemented with source of systemic testosterone to maintain human serum levels (∽4.0 ng/ml) 9, our group demonstrated that ADT-induced acute apoptotic death of the human prostate endothelial cells, and that endothelial cell apoptosis preceded the wave of epithelial cell apoptotic death by several days. Unexpectedly, the acute vascular involution in the primary xenografts was followed by a rapid restoration of the human vascular network as demonstrated by restoration of the endothelial cell linings of the prostate microvasculature to pre-ADT levels, in the absence of circulating androgens 10. The involution and rapid androgen-independent recovery of the microvascular endothelial cells in the human prostate tissue suggested activation of complex, tissue-specific mechanisms critical to the development of castration-recurrent growth and, therefore, may represent a new class of therapeutic targets that may be available transiently after ADT, providing a target tissue-specific ‘therapeutic window’.

Exploitation of platelets for targeted therapeutic intervention has been restricted to transfusion medicine, where platelets are a tool to control haemorrhage because of thrombocytopenia or iatrogenic immuno-suppression. However, the natural propensity for platelets to adhere to areas of endothelial damage suggested them as a prototype for development of multi-functional vehicles to target imaging/therapeutic agents selectively to the microvasculature of human prostate that was transiently perturbed by ADT during the ‘therapeutic window’. Fresh human platelets harvested from donors have a ‘shelf-life’ of 5 days, limiting their application to technologies that require *ex vivo* manipulation. However, mild aldehyde stabilization of fresh human platelets allows production of a lyophilized platelet product that when re-hydrated (hRL-P) retains near normal ultra-structure and exhibits functionality comparable to fresh platelets 11,12. In this report, human umbilical vein endothelial cells (HUVEC) cells *in vitro*, and short-term primary xenografts of human prostate tissue as an *in vivo* pre-clinical model, were utilized to investigate whether hRL-P demonstrated targeted binding to endothelial cells selectively activated/damaged *in vitro* by thrombin/ADP or human prostate endothelial cells perturbed *in vivo* by ADT. The successful *in vitro* and *in vivo* targeting of perturbed human endothelial cells suggested that engineered platelets represent a valuable prototype for development of multi-functional treatment vehicles to enhance therapeutic efficacy for treatment of prostatic disease.

## Material and methods

### Human prostate primary xenografts

Human prostate tissue was collected in accordance with National Institutes of Health guidelines on the use of human cases, with approval by the IRB at Roswell Park Cancer Institute (RPCI). Human prostate tissue samples were obtained from fresh radical prostatectomy remnants (within 2 hrs of resection) from at eight different patients. The majority of these male patients were over 55 years old with a median age of 68 years old and Gleason grades of the prostate cancers that varied between 3 + 3 and 4 + 3. None of the patients had undergone treatment for BPH or hormonal therapy prior to surgery. Prostate tissue was macro-dissected by a pathologist and designated as benign tissue (non-involved), or tumour (>70% cancer cells) tissue, using a recently reported protocol 13. After evaluation by the pathologist, tissue specimens were submerged immediately in ice-cold ViaSpan solution (Barr Laboratories Inc., Pomona, NY, USA), and transported on ice to the laboratory for transplantation. A specimen of the initial tissue (IT) remnant of at least 8 mm^3^ was removed from each surgical tissue sample before transplantation, fixed in 10% formalin, and paraffin-embedded for histological confirmation of the tissue as benign or malignant.

Primary xenografts of freshly harvested human prostate tissues were established in Severe Combined Immunodeficiency (SCID) mice as described previously 9,10,14. All experimental protocols that involved laboratory animals were performed in accordance with the National Institutes of Health guidelines and were approved by the Institutional Animal Care and Use Committee at RPCI. In brief, the tissue specimen was cut into wedge-shaped pieces 2–3 mm in length, and 2 mm in width at the broadest end, and the wedges were transplanted into male SCID mice, 3 months of age, that previously had been castrated and implanted subcutaneously with a 12.5 mg sustained-release testosterone pellet (Innovative Research of America, Sarasota, FL, USA) to maintain serum testosterone levels at ∽4.0 ng/ml throughout the study. For transplantation of prostate tissue, small (∽3 mm) incisions were made in the skin on the right and left flanks of immunocompromised mouse hosts anaesthetized with Domitor (1 mg/kg i.p.; Pfizer, Inc., New York, NY, USA), tissue wedges to be implanted dipped in Matrigel™ (BD Biosciences, Bedford, MA, USA), and the coated tissue wedges inserted into the subcutaneous space through a 10-gauge trocar device (Popper & Sons, Inc., Lincoln, RI, USA). Between 3 and 5 wedges were implanted along each flank through individual incisions; up to 10 fragments from a single patient were transplanted per animal. Incision sites were closed with Nexband tissue glue (Veterinary Products Laboratories, Phoenix, AZ, USA).

### hRLP adherence to vascular damage induced by androgen deprivation

Primary xenografts of human prostate tissue from control hosts (non-castrate) were harvested from animals pre-implanted with sustained-release testosterone pellets, maintained for 30 days, and administered platelets (hRL-P) by injection into the tail vein on day 30 after xenograft transplantation. Xenografts with microvasculature perturbed by ADT were harvested from animals pre-implanted with testosterone pellets, maintained for 30 days, ADT-initiated on day 30 (testosterone pellet removed) after transplantation with tissue, and administered hRL-P on day 1, 2 or 3 after initiation of ADT (castration). hRL-P were administered to living host animals 30 minutes before the animal was killed by exsanguination during perfusion to remove unbound platelets that had not adhered to the vasculature, and the xenografts harvested. Harvested xenografts were fixed in 10% formalin for a minimum of 24 hrs, after which the fixed tissues were paraffin-embedded. Paraffin blocks were sectioned (5.0 μm) onto ProbeOn Plus slides (Fisher Scientific International, Suwanee, GA, USA). Digital images of fields of the histological sections were collected, and the adhesion of hRL-P was quantitated as the area of the digital images occupied by platelets, as determined by CD42b immunostaining (Data S1), *versus* the total area of the xenograft tissue.

### Preparation of SPIO-RL platelets

Lyophilized human platelets were prepared as described 12. In brief, fresh platelets were incubated for 1 hr with paraformaldehyde (1.8%) and washed with citrated saline (0.006 M trisodium citrate/0.154 M NaCl, pH 6.8) with 5% bovine serum albumin. Platelets were lyophilized at −20°C to −40°C for 20–24 hrs. Dried platelets were stored at −80°C until used. Dried platelets were re-hydrated in 1.0 ml of imidazole buffer (IB; 0.084 M imidazole, pH 7.35) and centrifuged at 1000 × g for 8 min. to pellet the platelets. The re-hydrated platelets were freed of albumin and imidazole by three washes in citrated saline. For use, platelet pellets were resuspended in platelet-poor plasma (p2918, Sigma-Aldrich, St.Louis, MO, USA), or in a modified Hanks’ buffered salt solution (0.17 M NaCl/6.7 mM KCl/1.0 mM MgSO_4_/0.5 mM K_2_HPO_4_/2.8 mM Na_2_HPO_4_/13.8 mM dextrose, pH 7.2), for *in vitro* studies and in normal saline for *in vivo* studies.

Super-paramagnetic iron oxide nanoparticles (SPIOs: Feridex, Advanced Magnetics, Lexington, MA, USA), which were comprised of ∽5-nm SPIO cores with a dextran coating provided at an iron content of 11.2 mg Fe/ml 15, were diluted 1/10 with infusion-grade saline, combined with freshly harvested apheresis platelets at 1 × 10^9^/ml, and incubated overnight, which resulted in phagocytosis of the SPIOs by the platelets 16. After SPIO internalization, the iron nanoparticle-loaded platelets were separated from extracellular SPIO using size-exclusion chromatography, and the platelets were fixed with paraformaldehyde by the same procedures used for the production of hRL-Ps 12, to obtain SPIO-RL platelets.

### *In vitro and in vivo* binding of hRL platelets

hRL-P (2 × 10^5^ platelets/well) labelled with Green BODIPY or CMF2HC fluorescent dyes (Molecular Probes, Eugene, OR, USA) were used for visualization and quantification of specific binding *in vitro* of platelets to cultures of HUVEC that were activated with either thrombin (0.01–10 Units/ml) or ADP (adenosine 5′-diphosphate, 0.01–10 μM). For visualization and quantitation of platelet binding to human prostate endothelial cells perturbed by ADT, human prostate tissue xenografts transplanted to immunocompromised mouse hosts pre-implanted with slow-release pellets of testosterone were employed. Xenografts were maintained for 30 days on the host in the presence of human serum levels of testosterone. Control animals were treated with platelets on day 30 post-transplantation (androgen-stimulated or non-castrated). ADT treated animals had the testosterone pellet removed on day 30 post-transplantation, and animals were treated with platelets on day 1, 2 or 3 after ADT 10. At the time of analysis, animals were injected *i.v*. with hRL-P (10^6^ platelets/μl; 50 μl total volume), platelets allowed to circulate for 30 min., the vascular volume perfused with saline (3 ml) to remove unbound platelets, and the xenografts and select host tissues harvested for analysis of platelet localization. Prostate vasculature-bound platelets were visualized using immunohistochemistry analyses (CD42b immunostaining).

### Time of flight secondary ion mass spectroscopy (ToF-SIMS) analysis of xenografts of human prostate tissue incubated *in vivo* with SPIO-loaded platelets

ToF-SIMS experiments were performed on an ION ToF V 5–100 time of flight secondary ion mass spectrometer (ION ToF, gmbh), equipped with Bi, Cs and C_60_ primary ion beam sources operated in the high current bunched mode at 25 kV. The operation mode utilized for this analysis had an ultimate spatial resolution of 5–7 μm which is determined by the beam diameter of the Bi_3_^++^ primary ion gun on the ToF-SIMS instrument. A characteristic series of iron ions were identified to be present in isolated SPIO nanoparticles, in hRL-P loaded with SPIOs, and in xenografts tissues harvested from animals treated by IV injection with SPIO-hRL-P. The iron ions detected by ToF-SIMS were Fe, FeH, FeOH, FeO_2_H, FeH_3_O_4_ and Fe_2_O_8_H. The identification of the individual iron ion species was based on the theoretical mass of each iron containing ion as determined using the IonSpec® software program. The normalized level of iron in the xenograft tissue specimens was determined as the average intensity of five experimental spectra of either xenograft tissue from androgen-stimulated host animals or xenograft tissue harvested from animals on day 3 post-ADT.

## Results

### Binding of hRL platelets to primary cultures of human endothelial cells

**Figure 1:**
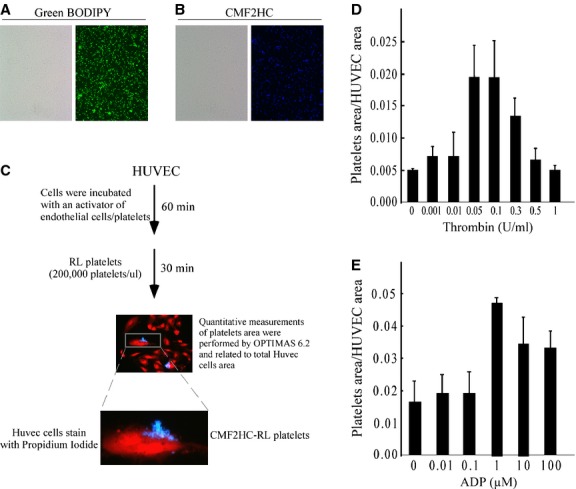
Adherence *in vitro* of hRL-P to HUVEC. (A and B) Visualization of hRL-P fluorescently labelled with CellTracker™ Green BODIPY® (A, right panel), and CMF2HC (B, right panel). Bright field image of the labelled platelets (A, B, left panels). (C–E) CMF2HC-labelled hRL-P adherence to HUVEC. (C) Experimental protocol for *in vitro* evaluation of adherence of RL-platelets to HUVEC in culture. (D and E) Dose–response curve for the effect of pre-activation of HUVEC by thrombin (D) or ADP (E) on adherence of CMF2HC-labelled hRL-P.

Initial experiments were focused on validation of the ability of fluorescently labelled hRL-P to adhere selectively *in vitro* to human endothelial cells that were pre-activated by exposure to increasing concentrations of thrombin or ADP. Thrombin, the key regulatory protein of haemostasis, is a potent stimulus for endothelial cell activation 17,18, thrombin-activated hRL-P demonstrated an intracellular stimulus response that was coupled through intracellular kinases 19 and hRL-P degranulated in response to ADP during fibrin clot formation 20. hRL-Ps covalently labelled with Green BODIPY (Fig.1A) or CMF2HC (Fig.1B) dyes were used for visualization and quantification of specific binding to activated HUVEC in culture (Fig.1C). In four independent experiments, activation of HUVEC using either, thrombin or ADP, induced marked increases in adherence of hRL-P (Fig.1D and E). Thrombin-induced adherence increased in a dose-dependent manner, peaking at a thrombin exposure of 0.1 U/ml, and decreasing at higher concentrations to levels comparable to un-stimulated HUVEC controls (Fig.1D). This dose–response curve for platelet adherence was similar to published observations for thrombin-induced endothelial cell inter-cellular gap-formation and erythrocyte adhesion, including the curve shape and maximum concentration 17. In contrast, ADP-induced adherence of hRL-Ps to activated HUVEC peaked at an ADP concentration of 1.0 μM, and the maximal response plateaued at ADP concentrations between 1.0 and 100 μM. These *in vitro* studies validated previous reports that hRL-P retained the native primary haemostatic function of native platelets 12. Moreover, fluorescent labelling of hRL-Ps did not degrade their central biological activity of binding to activated endothelial cells.

### Optical Imaging of *in vivo* binding of hRL-P to damage human vasculature in primary xenografts of prostate tissue

**Figure 2:**
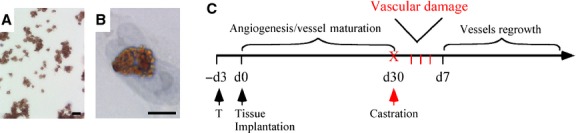
*In vitro* and *in vivo* tools to study selective binding of hRL-P to damaged human vascular network induced by androgen deprivation. (A and B) Immuno-cytochemical identification of hRL-P visualized by CD42b immuno-histochemical labelling of RL-platelets alone (A) or after co-incubated with activated HUVEC (B). (C) Diagram illustrating the time course for studies in the pre-clinical model of primary xenografts of human prostate tissue; bar: 5 μM.

Fluorescence labelling of RL platelets before injection into animal hosts offered many potential advantages for analysis of platelet localization in histological sections prepared from human prostate. However, prostate tissue is notable for extremely high levels of auto-fluorescence. Consequently, in contrast to the immunofluorescence-based visualization used for the *in vitro* studies, immuno-histochemical analysis based upon a monoclonal antibody specific for a human platelet marker, CD42b, was utilized for visualization and localization of RL platelets in the human prostate xenografts and host mouse tissues. CD42b is a platelet-specific glycoprotein (GPIb) that serves as a receptor for von Willebrand factor, and it is a high-affinity receptor for thrombin. As proof-of-concept, immuno-histochemical staining with anti-CD42b identified both free RL platelets and RL platelets adherent to activated HUVEC (Fig.2A and B).

Primary xenografts of intact human prostate tissue provide a unique system for characterization of the targeted binding of human platelets to human vasculature acutely damaged by androgen deprivation. Primary xenografts of fresh surgical tissue recapitulate both the tissue architecture of the intact human prostate tissue and the prostate-specific vascular response to ADT. Microvessel density in xenografts of both benign prostate tissue and prostate cancer tissue decreased acutely after ADT, and rebounded to pre-castration levels between days 7–14 post-ADT, in the continued absence of testosterone 10 (Fig.2C).

**Figure 3:**
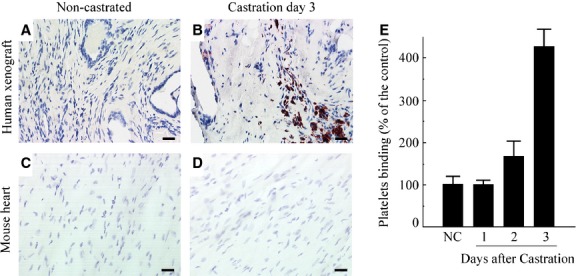
*In vivo* binding of hRL-P to ADT-damaged human vasculature in primary xenografts of prostate tissue. CD42b immuno-histochemical labelling of hRL-P in human prostate xenografts (A and B) and mouse heart (C and D) from control (non-castrate) mice (A and C) and mice on the day 3 following androgen deprivation (B and D). At the time of analysis, animals were injected *i.v*. with RL-platelets (10^6^ platelets/μl), incubated for 30 min. prior to euthanization by exsanguination during perfusion to remove unbound platelets, and xenograft harvested; bar: 20 μM. (E) Quantification of the binding of hRL-P to the human prostate xenografts. Values in xenografts from hosts incubated with platelets on day 1–3 post-ADT were expressed as the per cent of platelets that adhered to xenografts in control hosts (NC; represents average of four independent experiments).

**Figure 4:**
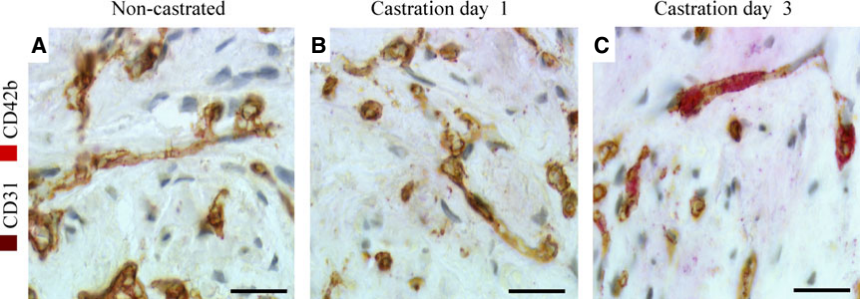
hRL-P co-localized with human prostate endothelial cells damaged by androgen deprivation in xenografts of human prostate tissue. (A–C) Immuno-histochemical co-staining of endothelial cells with anti-human CD31 (brown) and platelets with anti-human CD42b (red) in human prostate xenografts from control mice (non-castrated) and xenografts from mice treated with platelets on days 1 and 3 following androgen deprivation; bar: 20 μM.

Analysis of the ADT-induced binding of hRL-Ps to human prostate endothelial cells selectively damaged by ADT revealed a marked increased in adherence of hRL-P within prostate xenografts treated with platelets on day 3 post-initiation of ADT relative to xenografts from androgen-stimulated host mice treated with platelets in a normal androgenic environment (Fig.3A and B). From a clinical/therapeutic perspective, a critical question was whether hRL-P targeted predominantly the transiently damaged human prostate vasculature, with minimal binding to endothelial cells in an intact androgenic environment or in tissues. Selective binding to only the prostate vasculature would minimize collateral morbidity after systemic administration of multi-functional therapeutic vehicles. Tissue harvested from the heart (Fig.3C and D), liver, lungs, spleen and intestine (data not shown) of host control mice, or mice exposed to platelets after ADT, revealed no evidence of hRL-P localization to the microvasculature of host organs. Quantitation of the area occupied by platelets in the post-ADT human prostate xenograft specimens relative to the area occupied by platelets in control human prostate xenograft specimens showed increased adherence of hRL-P in the xenografts on the day 3 after ADT (Fig.3E). To verify the targeting of hRL-P was to the endothelial cells of the transiently damaged human prostate vasculature in the ADT-perturbed xenografts, visualization of localization of hRL-P in the xenografts was performed with dual immuno-histochemical staining for endothelial cells (anti-CD31) and platelets (anti-CD42b). hRL-P (red stain) were demonstrated to co-localize with human prostate endothelial cells (brown stain) only on day 3 post-ADT; hRL-P were not observed to localize in the microvasculature of the prostate xenografts from control animals (non-ADT) injected with platelets, or animals injected with platelets on day 1 post-ADT (Fig.4A–C). These results confirmed that platelets modulated *ex vivo* by loading with SPIOs retained the haemostatic properties necessary for *in vivo* haemostasis.

### ToF-SIMS chemical images of *in vivo* binding of bioengineered hRL-P to damage human vasculature in primary xenografts of prostate tissue

**Figure 5:**
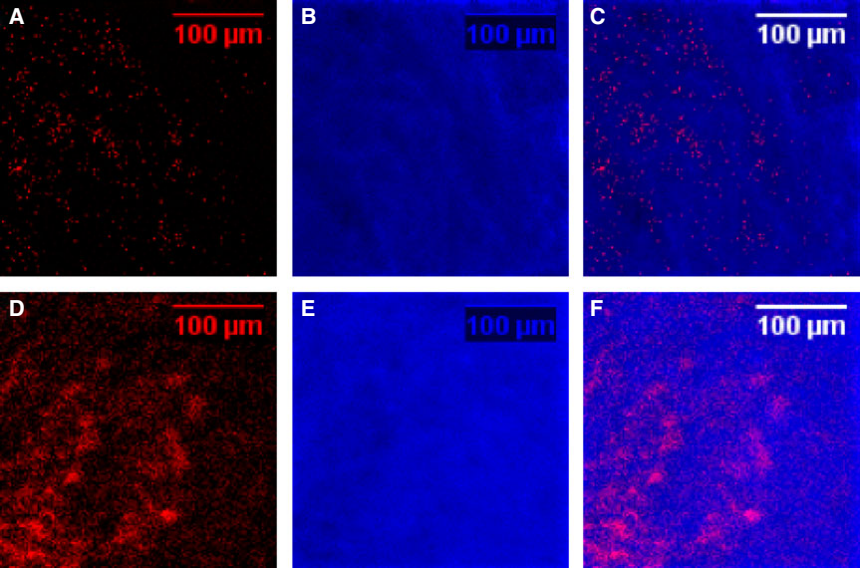
Human platelets loaded *ex vivo* with SPIO nanoparticles maintain their ability to target specifically the ADT-labilized human prostate vasculature. Pseudo-colour represents iron levels based on ToF-SIMS chemical images of xenograft tissue from control animals (non-castrate; A–C) and xenograft tissue from animals from day 3 after initiation of ADT (D–F). (A and D) Red pseudo-colour represents the summed intensity for all iron ion species associated with SPIO. (B and E) Blue pseudo-colour represents the intensity of the control phosphocholine ion. (C and F) Overlay of pseudo-colour representations of levels of iron ions and phosphocholine. Normalization represents five experimental spectra for xenograft tissue harvested from both non-castrate controls and from day 3 post-ADT host.

Human re-hydrated lyophilized platelets loaded with SPIO nanoparticles before fixation and lyophilization were employed as a proof-of-concept to demonstrate that exogenously bioengineered platelets could serve as a prototype for multi-functional therapeutic vehicles, and could provide a tool for evaluation of the PK/PD characteristics of vehicles that can be targeted specifically to transiently destabilized human prostate vasculature, or possibly the unstable vasculature of tumours. The iron component of the SPIO nanoparticles pre-loaded into the platelets provided a unique tool for validation of organ-specific localization of platelets by identification by ToF-SIMS analysis of the presence of characteristic Fe ions. ToF-SIMS relies on the capacity of the iron to be readily ionizable, and the ions identified in Fig.5 as present in the xenograft tissue on day 3 after castration were identical to those that were identified in the static spectra of SPIO-hRL-P alone. The small mass deviation (±20 ppm) between the experimentally observed and theoretical masses of the Fe ions demonstrated that iron ions from SPIO nanoparticles localized in hRL-Ps were the result of the adherence to the ADT-perturbed xenograft vasculature of hRL-P pre-loaded with SPIO (Table S1). Chemical imaging analysis (ToF-SIMS) allowed determination of the relative quantity and crude spatial distribution of SPIO nanoparticles in xenograft tissue samples (Figure5 and Table1). The images in Figure5 show the distribution of the SPIO iron ions in xenograft tissue from animals from control (non-castrate; Fig.5A–C) *versus* in xenograft tissue on day 3 after initiation of ADT, correlated with adherence of SPIO-hRL-P (Fig.5D–F). The SPIO iron ion signal in the chemical image of the xenografts harvested on day 3 after castration was significantly higher than in the chemical image of the matched control, and the spatial localization of the signals suggests aggregates of platelets, consistent with the IHC observations in histological specimens. Analysis of the relative intensity of the SPIO iron ions in the xenografts from control hosts *versus* xenografts from hosts after ADT demonstrated approximately a 200-fold increase in SPIO in the xenografts harvested from hosts on day 3 post-ADT (Table1). This data, coupled with the imaging data, demonstrated that mild aldehyde stabilization of fresh platelets, or *ex vivo* loading of platelets with SPIO nanoparticles, allowed production of a lyophilized platelet product that when re-hydrated (hRL-P) exhibited targeting comparable to fresh platelets.

**Table 1:** Quantitation of relative intensity of pseudo-colour representations of iron levels based on ToF-SIMS chemical images of xenograft tissue

Xenograft tissue	Average normalized intensity (sum iron ions/C_5_H_15_PNO_4_)	SD
Non-castrated	4.0 × 10^−3^	±5.0 × 10^−4^
Castration day 3	0.8	±0.3

## Discussion

Although cancer cells are inherently more vulnerable than normal cells to the effect of chemotherapy agents, particularly drugs that target highly proliferative cells, these drugs are non-selective and cause collateral injury to normal tissues. Therefore, during the last decade efforts have been made to develop novel carriers for both existing and new drugs that more specifically target the tumour cell and its microenvironment. In this regard, the use of nanoparticles in the diagnosis, treatment, and imaging in early cancer is emerging as a powerful tool. However, the main challenge remains, developing reagents that target specifically a component of the tumour, enhancing delivery to the tumour while minimizing collateral toxicity to normal tissues. As a result of the remarkable increase in tumour vasculature during cancer progression, and the striking morphological differences between tumour vasculature and normal blood vessels, tumour vessels represent a largely unexplored primary target for tumour-specific delivery. Unlike the normal vasculature, blood vessels within tumours are leaky, tortuous, dilated and the endothelial cells have aberrant morphology. As a result of the absence of a functional basement membrane, the tumour vessels also are hyper-permeable and pericytes/mural cells are loosely attached or entirely absent 3,21. These abnormalities in tumour vessels define a pro-thrombotic environment capable of platelet activation that should be absent from the rest of the vascular network. Furthermore, we propose a prostate-specific mechanism to enhance further targeting the perturbed human prostate vasculature by selectively inducing endothelial cell apoptosis by short-term androgen deprivation. Consequently, we propose that ADT produces a transient ‘therapeutic window’ that allows targeted delivery of multi-functional nanoparticle vehicles to prostate cancer tissue based on the physiological predisposition of platelets to adhere to areas of endothelial damage. This unique approach will facilitate paradigm changing imaging and therapeutic options that will enhance therapeutic efficacy after systemic administration while minimizing collateral morbidity.

The platelet is a logical vehicle for targeting delivering to the innately or therapeutically (ADT) destabilized tumour vasculature in human prostate cancer. However, the logistical problem of harvesting, storing and manipulating *ex vivo* autologous platelets, particularly from elderly prostate cancer patients, renders this application difficult, although autologous platelet administration is a common clinical protocol. Because of the short ‘shelf-life’ of platelets, considerable efforts have been made to apply the process of freeze-drying to platelets, including platelets manipulated *ex vivo*, to provide a stable infusible haemostatic agent 12,22. The development of a technique by Entegrion, Inc to lyophilize platelets after mild aldehyde stabilization, produced a product that when re-hydrated (RL platelets) retained a near normal ultra-structure and exhibited the functionality of fresh platelets, including the capability of degranulation upon activation and recruitment of additional platelets 11,12,22. Furthermore, *in vivo* studies using balloon-denuded coronary arteries in dogs demonstrated that RL platelets mediated haemostasis at sites of vascular injury 12. Localization of RL platelets to wound sites also was demonstrated in studies involving pigs with severe haemorrhagic shock because of washout thrombocytopenia 23. Our report is the first study to demonstrate that RL platelets can be used to deliver therapeutic and/or imaging modalities in pre-clinical models involving human vascular injury or tumour vasculature.

The hypothesis to utilize bioengineered platelets as multi-functional nanodevices for delivery of imaging or therapeutic agents was motivated by the critical need to improve current imaging technologies and standard-of-care for prostate cancer patients. Our laboratory developed a pre-clinical model of primary xenografts of fresh surgical specimens of human prostate transplanted to immunocompromised hosts that were pre-implanted with sustained-release testosterone pellets to provide human serum levels of circulating androgen. This pre-clinical model system recapitulates the tissue architecture and androgen-regulated homoeostasis of the intact human prostate, the vasculature in the xenografts is of human origin, and androgen deprivation induces acute endothelial cell death and human vascular involution 9,10. In this report, we demonstrate that ADT-induced selective damage to the vasculature in human prostate elicits binding of exogenously added bioengineered human platelets, without inducing binding to vasculature in organs of the immunocompromised host.

Previous studies have demonstrated that platelets internalize certain forms of SPIO nanoparticles in a robust manner 16. Utilizing primary cultures of human endothelial cells, and the short-term primary xenografts of human prostate, this study supports a novel concept that human platelets loaded with imaging or treatment modalities can be used as ‘Trojan Horses’ during the ‘therapeutic window’ opened by the vascular perturbation induced in the human prostate by ADT. This alternative paradigm provides an approach to selectively destabilize the microvascular network of the prostate, facilitating elimination of multi-focal malignant disease processes by targeting a cell type that should maintain a more consistent phenotype than the heterogeneous tumour cell population.

## Supporting Information

**Data S1.** Supplemental experimental procedures. **Table S1.** Summary of mass accuracy analysis by TOF-SIMS.

## References

[b1] Folkman J (1971). Tumor angiogenesis: therapeutic implications. N Engl J Med.

[b2] Ebos JM, Kerbel RS (2011). Antiangiogenic therapy: impact on invasion, disease progression, and metastasis. Nat Rev Clin Oncol.

[b3] Jain RK (2005). Normalization of tumor vasculature: an emerging concept in antiangiogenic therapy. Science.

[b4] 1Vartanian RK, Weidner N (1995). Endothelial cell proliferation in prostatic carcinoma and prostatic hyperplasia: correlation with Gleason’s score, microvessel density, and epithelial cell proliferation. Lab Invest.

[b5] Weidner N, Carroll PR, Flax J (1993). Tumor angiogenesis correlates with metastasis in invasive prostate carcinoma. Am J Pathol.

[b6] Lissbrant IF, Stattin P, Damber JE (1997). Vascular density is a predictor of cancer-specific survival in prostatic carcinoma. Prostate.

[b7] Alonzi R, Padhani AR, Taylor NJ (2011). Antivascular effects of neoadjuvant androgen deprivation for prostate cancer: an *in vivo* human study using susceptibility and relaxivity dynamic MRI. Int J Radiat Oncol Biol Phys.

[b8] Kravchick S, Cytron S, Mamonov A (2009). Effect of short-term dutasteride therapy on prostate vascularity in patients with benign prostatic hyperplasia: a pilot study. Urology.

[b9] Montecinos VP, Godoy A, Hinklin J (2012). Primary xenografts of human prostate tissue as a model to study angiogenesis induced by reactive stroma. PLoS ONE.

[b10] Godoy A, Montecinos VP, Gray DR (2011). Androgen deprivation induces rapid involution and recovery of human prostate vasculature. Am J Physiol Endocrinol Metab.

[b11] Fischer TH, Bode AP, Parker BR (2006). Primary and secondary hemostatic functionalities of rehydrated, lyophilized platelets. Transfusion.

[b12] Read MS, Reddick RL, Bode AP (1995). Preservation of hemostatic and structural properties of rehydrated lyophilized platelets: potential for long-term storage of dried platelets for transfusion. Proc Natl Acad Sci USA.

[b13] Morrison C, Cheney R, Johnson CS (2009). Central quadrant procurement of radical prostatectomy specimens. Prostate.

[b14] Gray DR, Huss WJ, Yau JM (2004). Short-term human prostate primary xenografts: an *in vivo* model of human prostate cancer vasculature and angiogenesis. Cancer Res.

[b15] Clement O, Siauve N, Cuenod CA (1998). Liver imaging with ferumoxides (Feridex): fundamentals, controversies, and practical aspects. Top Magn Reson Imaging.

[b16] Oldenburg AL, Gallippi CM, Tsui F (2010). Magnetic and contrast properties of labeled platelets for magnetomotive optical coherence tomography. Biophys J.

[b17] Manodori AB, Matsui NM, Chen JY (1998). Enhanced adherence of sickle erythrocytes to thrombin-treated endothelial cells involves interendothelial cell gap formation. Blood.

[b18] Garcia JG (1992). Molecular mechanisms of thrombin-induced human and bovine endothelial cell activation. J Lab Clin Med.

[b19] Fischer TH, Merricks EP, Russell KE (2000). Intracellular function in rehydrated lyophilized platelets. Br J Haematol.

[b20] Fischer TH, Merricks EP, Bode AP (2002). Thrombus formation with rehydrated, lyophilized platelets. Hematology.

[b21] Dvorak HF (2003). Rous-Whipple Award Lecture. How tumors make bad blood vessels and stroma. Am J Pathol.

[b22] Bode AP, Fischer TH (2007). Lyophilized platelets: fifty years in the making. Artif Cells Blood Substit Immobil Biotechnol.

[b23] Fischer TH, Merricks E, Nichols TC (2001). The co-infusion of rehydrated, lyophilized platelets with HBOC-201 for hemostasis in dilutional thrombocytopenia. Blood.

