# Supplementary material for: Selective targeting of bioengineered platelets to prostate cancer vasculature: new paradigm for therapeutic modalities
Source: J Cell Mol Med. 2015 Mar 4;19(7):1530–7. doi: 10.1111/jcmm.12515 (PMC4511351; doi:10.1111/jcmm.12515)
Supplement: Supplementary file 1 [file jcmm0019-1530-sd1.doc]

**Selective targeting of bioengineered platelets to prostate cancer vasculature: new paradigm for therapeutic modalities**

**Supplemental information**

**Immunostaining Analysis**

Histological specimens were prepared from each IT specimen harvested from surgical prostate cancer tissue specimens before implantation, and from all corresponding xenograft specimens. Tissue sections were de-paraffinized using Citrasolv (Fisher), hydrated through graded washes of ethanol and de-ionized water, and equilibrated in automation buffer (Biogenex, San Ramon, CA). Antigens were retrieved by boiling the slides in 10.0 mM citric acid buffer (pH 6.0). Endogenous peroxidase activity was blocked before incubation with primary antibody. After blocking, tissue sections were incubated overnight with antibodies against human CD31 (huCD31: 1:40, Dako, Carpinteria, CA), huCD34 (1:100, Neomarkers, Fremont, CA) and CD42b (1:100, Millipore, Billerica, MA). All antibodies were diluted in 10.0 mM Tris-HCl buffer (pH 7.8) that contained 8.4 mM sodium phosphate, 3.5 mM potassium phosphate, 120 mM NaCl, and 1.0 % BSA (w/v). After incubation with primary antibody, tissue sections were washed 3 times in Tris-HCl buffer (pH 7.8) for 10 min each, and incubated with HRP-conjugated anti-rabbit IgG or anti-mouse IgG (1:100, DakoCytomation) for 2 h at room temperature. Peroxidase activity was developed using 100 mM Tris-HCl buffer that contained 3,3-diaminobenzidine tetra-hydrochloride (1.0 μg/ml, Sigma-Aldrich) and H_2_O_2_ (1.0 μl/ml, VWR International, West Chester, PA). Hematoxylin (Harris) was used as a nuclear counter-stain in tissue sections. Stained slides were dehydrated by sequential steps through a graded series of alcohol washes and Citrisolv (Fisher, Suwanee, GA) and were mounted using coverslips. For immuno-fluorescence studies, after incubation with primary antibody the specimens were incubated for 2 h with AlexaFluor488- or AlexaFluor594-conjugated affinity-purified donkey anti-rat or anti-mouse IgG (1:200, Molecular Probes, Eugene, OR) secondary antibody at room temperature. DAPI (4’,6’-diamidino-2-phenylindole dihydrochloride) was used to counterstain nuclei for immunofluorescence studies. IHC or immunofluorescence staining in the absence of primary antibody, or using pre-immune serum, provided negative controls.

**Digital Image Collection and Analysis**

Digital images of immuno-histochemically stained sections were collected using a Kontron ProgRes 3012 camera (TriPath Imaging, Inc., Burlington, NC) mounted on an Axioskop microscope (Carl Zeiss, Inc., Thornwood, NY) at 100x, 200x, 400x and 630x magnification, depending on the intended use of the images. Image analysis for manual counts of objects was performed using ImageJ software with the Cell Counter Plug-In (Research Services Branch, National Institute of Mental Health, Bethesda, MD). Three 100x (0.480 mm2), 200x (0.120 mm2) or 400x (0.030 mm2) fields were collected and analyzed. Digital images of immuno-histochemically stained sections were collected using a Hamamatsu Color Chilled 3CCD camera (Hamamatsu, Bridgewater, NJ) mounted on an Axioskop microscope (Carl Zeiss) at total magnification 400x.

**ToF-SIMS Analysis and Determination of the Relative Intensity of SPIO in the Xenografts Tissues**

ToF-SIMS (Time of Flight-Secondary Ion Mass Spectroscopy) experiments were performed on an ION TOF V5-100 time of flight secondary ion mass spectrometer (ION TOF gmbh) equipped with Bi, Cs, and C_60_ primary ion beam sources. The Bi_3_^++^ primary ion beam was utilized as the analysis gun for both static and imaging analysis in this study and was operated in the high current bunched mode at 25kV. Variation in the analysis area occurred during sample analysis, therefore, the primary ion dose varied per raster area. The target current was measured by a Faraday cup to be ~0.3 pA. Each raster area was divided into 128 x128 pixels, and analyzed in the positive ion mode with a cycle time of 120 ms. The scale bar on each image was drawn using ImageJ. The mass analyzer was operated in the high mass resolution mode where the secondary ions were detected by a microchannel plate detector with a post-acceleration energy of 10 kV. Frozen, hydrated samples were analyzed using a cold stage installed on the ION TOF V. Samples were cooled to -125^o^C and were kept at this temperature throughout analysis.

**Mass Accuracy Analysis and Determination of the Relative Intensity of SPIO in the Xenografts Tissues**

Determination of mass accuracy was done using the average mass of the iron containing ions identified in a sample of human rehydrated platelets and SPIO (Superparamagnetic iron oxide) nanoparticles in PBS. For ToF-SIMS analysis, the sample was vacuum dried onto a single crystal 1 cm^2^ silicon substrate. The iron component of the parent SPIO nanoparticle was used to determine the characteristic ions since iron is readily ionizable in a ToF-SIMS instrument. The ions in Table 1 represent those that were identified in static a spectra of SPIO RL platelets alone, and all species were identified subsequently in xenografts tissue specimens containing SPIO loaded platelets. The theoretical mass of each iron containing ion was determined using the IonSpec® software program. All calculations were done using Microsoft Excel and the deviation from the theoretical mass is reported in parts per million (ppm). The small mass deviation (+/- 20 ppm) between the experimental and theoretical masses demonstrates the detected iron ions were associated with the SPIO RL platelets within the xenograft tissue. The determination of the relative intensity of SPIO in the xenograft tissue harvested from non-castrated (control) hosts versus the androgen-deprived hosts at Day 3, was done by a normalization analysis. The normalization was completed by first summing the total intensity of the iron ions identified as due to the SPIO. This summed intensity was then divided by the intensity of the phosphocholine ion (C_5_H_15_PNO_4_^+^) in that spectrum. The phosphocholine ion is associated with the cell membrane that is observed routinely in ToF-SIMS mass spectra of biological samples^1, 2^ and was present at high intensity in the xenografts tissues allowing use as a control for normalization of the amount of tissue.

Supplementary Table 1. Summary of mass accuracy analysis. The actual mass is the average of that ion collected in five independent experimental spectra.

| Ion | Theoretical Mass | Actual Mass | Deviation |
| --- | --- | --- | --- |
| Fe | 55.9 | 55.9 | 1.8 |
| FeH | 56.9 | 56.9 | 8.0 |
| FeOH | 72.9 | 72.9 | 5.0 |
| FeO_2_H | 88.9 | 88.9 | 15.4 |
| FeH_3_O_3_ | 106.9 | 106.9 | 4.2 |
| FeH_3_O_4_ | 122.9 | 122.9 | -3.0 |
| Fe_2_O_8_H | 240.8 | 240.8 | -18.2 |

**1.** Sjovall P, Lausmaa J, Nygren H, Carlsson L, Malmberg P. Imaging of membrane lipids in single cells by imprint-imaging time-of-flight secondary ion mass spectrometry. Anal Chem 2003;75:3429-34.

**2.** Brunelle A, Touboul D, Laprevote O. Biological tissue imaging with time-of-flight secondary ion mass spectrometry and cluster ion sources. J Mass Spectrom 2005;40:985-99.
